# A Four-Step Platform to Optimize Growth Conditions for High-Yield Production of Siderophores in Cyanobacteria

**DOI:** 10.3390/metabo13020154

**Published:** 2023-01-20

**Authors:** Karishma Kundu, Roberta Teta, Germana Esposito, Mariano Stornaiuolo, Valeria Costantino

**Affiliations:** 1 The Blue Chemistry Lab, Department of Pharmacy, University of Naples Federico II, Via Domenico Montesano 49, 80131 Napoli, Italy; 2 Department of Pharmacy, University of Naples Federico II, Via Domenico Montesano 49, 80131 Napoli, Italy

**Keywords:** schizokinen, synechobactin, *Anabaena flos aquae*, biosynthesis of marine drugs, cyanobacteria, iron-chelating molecules, natural products, dereplication strategy, molecular network

## Abstract

In response to Iron deprivation and in specific environmental conditions, the cyanobacteria *Anabaena flos aquae* produce siderophores, iron-chelating molecules that in virtue of their interesting environmental and clinical applications, are recently gaining the interest of the pharmaceutical industry. Yields of siderophore recovery from in vitro producing cyanobacterial cultures are, unfortunately, very low and reach most of the times only analytical quantities. We here propose a four-step experimental pipeline for a rapid and inexpensive identification and optimization of growth parameters influencing, at the transcriptional level, siderophore production in *Anabaena flos aquae*. The four-steps pipeline consists of: (1) identification of the promoter region of the operon of interest in the genome of *Anabaena flos aquae*; (2) cloning of the promoter in a recombinant DNA vector, upstream the cDNA coding for the Green Fluorescent Protein (GFP) followed by its stable transformation in *Escherichia Coli*; (3) identification of the environmental parameters affecting expression of the gene in *Escherichia coli* and their application to the cultivation of the *Anabaena* strain; (4) identification of siderophores by the combined use of high-resolution tandem mass spectrometry and molecular networking. This multidisciplinary, sustainable, and green pipeline is amenable to automation and is virtually applicable to any cyanobacteria, or more in general, to any microorganisms.

## 1. Introduction

Siderophores are low-molecular-weight iron-chelating molecules secreted by microorganisms in response to iron deprivation and responsible for extracellular binding and intracellular uptake of ferric ions [1]. Almost all known bacterial species produce siderophores, which represent the molecules mostly involved in iron scavenging in the microbial world [2]. Several microorganisms can also uptake siderophores from neighbouring species (xenosiderophores) and pressuring producers to biosynthesise new siderophores.

The need to survive in habitual low iron conditions (lakes, rivers, and oceans present iron in nM concentration [3]) has led to the development of these excellent iron chelators, each endowed with peculiar structural features. Many siderophore structures have been isolated from cyanobacteria, with schizokinen from the cyanobacteria *Anabaena* sp. (PCC-7120) being the first one reported [4]. The overall structure of cyanobacterial siderophores can vary among species; however, the functional groups that chelate ferric iron are relatively conserved and include hydroxamates [5], catecholate [6], carboxylates and alfa-hydroxy-carboxylates, all featuring two oxygen donor atoms incorporated in either linear or cyclic structure [7]. Some siderophores are membrane-anchored amphiphilic molecules, a chemical feature that allows limited diffusion of siderophore–iron complexes [8]. 

In virtue of their potential environmental and clinical application, siderophores are recently gaining the interest of the pharmaceutical industry. As environmental tools, siderophores could find immediate application in protecting agricultural and aquaculture from metal pollution [9]. As pharmaceuticals, siderophores are effective in inhibiting metalloenzymes [10] and exert an atheroprotective effect in humans [11]. Siderophore-rich functional foods and food additives are under investigation in virtue of their efficacy in reducing iron overload (and the consequent oxidative stress), commonly occurring in people affected by cardiovascular diseases [11,12]. Recently, siderophore-conjugated antibiotics have been under testing to upgrade anti-infective therapy [13].

The different industrial fields interested in siderophores suffer, however, from the low yields of siderophore recovery from in vitro producing microbial cultures. Indeed, most of the times, the yield of siderophore produced in vitro achieves barely analytical quantities. Notwithstanding, yields are greatly improved upon optimization of the microbial growth conditions [14]. Till now, the identification of the ideal physical/chemical parameters for optimal siderophore production has been achieved using the One Strain Many Compounds (OSMAC) protocol [15]. The latter consists in the testing of different growth media to cultivate the siderophore-producing specific strain. Small-scale cultures (20 to 50 mL in volume) are ultimately compared in terms of final yield of siderophore production. The procedure is, however, expensive and time consuming (siderophore production in cyanobacteria requires 30 days of culturing before harvesting) and not suitable for high throughput screening and automation. 

We here propose an alternative experimental pipeline to rapidly identify growth parameters influencing and boosting siderophore production. The rationale of this four-step pipeline relies on the fact that: (i) the enzymes responsible for siderophore biosynthesis are most of the time clustered in a unique operon and are thus under the control of a single promoter, and that (ii) these operons are tightly regulated at the transcriptional levels and controlled in many ways (iron availability, product negative feedback, substrate availability, temperature, pH, media composition, culture crowding). The four-step pipeline includes: (1) identification of the promoter region of the operon of interest in the genome of the microorganism; (2) cloning of the promoter in a recombinant DNA vector, upstream of the cDNA coding for the Green Fluorescent Protein (GFP) followed by its stable transformation in *Escherichia coli*; (3) identification of the growth parameters affecting expression of the gene in *Escherichia coli* and their application to the cultivation of the *Anabaena* strain; (4) analyses of the cultures by extraction with organic solvents and dereplication by the combined use of high-resolution tandem mass spectrometry with molecular networking. 

With the aim to test our platform, we here describe its application in identifying growth parameters and environmental conditions affecting at the transcriptional level the production of siderophores such as schizokinen, synechobactin A by the cyanobacteria *Anabaena flos aquae* UTEX1444 (synonym, *Anabaena variabilis* Kutzing, *Anabaena variabilis* ATCC 29413, *Thrichormus variabilis* ATCC 29413, etc.) [16,17,18]. The results of the screening allowed the identification of temperature, pH, osmolarity, as well as iron and citrate concentrations as factors affecting, at the transcriptional level, siderophore production. Similarly, the analysis of the siderophore promoter allowed the identification of an unprecedented citrate responsive element affecting transcription of gene involved in siderophore production in *Anabaena flos aquae*.

To validate the pipeline, the identified growth parameters are here confirmed in in vitro cultures of *Anabaena flos aquae* UTEX 1444, with their extracts analysed and siderophores identified using our dereplication strategy based on the LC-HRMS based molecular networking [19].

## 2. Materials and Methods

### 2.1. Materials

Bactotryptophan (code 91079-40-2); Bacto yeast (code 8013-01-2); Sodium Chloride (code 7647-14-5); Agar (code 9002-18-0); Calcium Chloride (code 10035-04-8); Isopropanol (code 67-63-0); Ethanol (code 64-17-5); SSC Buffer (code 6135-04-3); Hind III (code 81295-22-9); BGL II (code 81295-12-7); Ammonium Citrate Tribasic (code 3458-72-8); Citric Acid (code 77-92-9); Lysine (code 56-87-1); Ferrous Sulphate (code 7720-78-7) were all from Sigma Aldrich. Glycerol (code 56-81-5); Midiprep Kit, (code K0841); Agarose Gel (code 9012-36-6); Propidium Iodide (code 25535-16-4); DNA Digestion Kit (code AM1907); Ligation Buffer (code IVGN2104) were from Thermo Fischer Scientific. Ampicillin (code 69-52-3) and Tris Acetate Buffer (code 135852-26-5) were from Fischer Scientific; Blue-Orange Loading Dye was from Promega; Miniprep Kit was from Euro genomics and Ferric Chloride (code 7705-08-0) was from Merck Millipore. 

### 2.2. Anabaena flos Aquae Genome Analyses

The genome of *Anabaena variabilis* ATCC 29,413 was retrieved from GenBank (Accession: CP000117.1, National Library of Medicine, NIH) [20]. The *Iuc* operon was analysed by means of Operon Mapper [21] to identify operon coordinates, operon–gene pairs, ORF coordinates and predict gene and protein sequences. The promoter region of the *Iuc* operon was analysed by using the software BacPP [22]. 

### 2.3. Bacterial Cultures

*Anabaena flos aquae* UTEX 1444 gifted by prof. Antonio Pollio (Department of Biology, Unina, Italy) was cultured in FW BG11 medium (freshwater BG11, 1L of the solution contains: Na_2_EDTA—1 mg, Citric Acid—6 mg, NaNO_3_- 500 mg, K_2_HPO_4_3H_2_O- 40 mg, MgSO_4 ×_ 7H_2_O- 75 mg, CaCl_2_- 26.4 mg, NaCO_3_- 17.1 mg, NiSO_4_ (NH_4_)_2_ SO_4_6H_2_O—250 µL of 0.1 mM stock solution, Na_2_SeO_4_—100 µL of 0.1 mM stock solution, Nitsch Solution—1 mL (where 100 mL solution contains: concentrated H_2_SO_4_—0.5mL, MnSO_4_.H_2_O—2.29 mg, ZnSO_4_.7H_2_O—0.5 g, CuSO_4_.5H_2_O—15.9 mg, Na_2_MoO_4_.2H_2_O—0.025 g, H_3_BO_3_—0.5 g, and, CoCl_3_.6H_2_O—0.135 g)) fortified with vitamin mix (100 mL of 10X solution contains: nicotine acid—100 mg, PABA—10 mg, biotin—1 mg, thiamine—251 mg, vitamin B12—1 mg, folic acid—1mg, inositol—1 mg, Calcium-pantothenate—100 mg). Cultures were grown in culture media with six different concentrations of citrate supplemented as sodium citrate. The concentration of sodium citrate was determined by making a series of dilutions from a 100 mM parent solution to get the following concentrations, such as 0 mM (#1); 0.1 mM (#2); 1 mM (#3); 10 mM (#4); 100 mM (#5); and Standard FW BG11 STD (#6). Then, a second serial dilution was made for cultures supplemented of ferric ammonium citrate (concentration from 0 µM to 5 µM). Triplicates of all cultures were cultivated at the temperature of 29˚C for 30 days. 

*E. coli* was grown either in Luria Broth (Bacto tryptone—10 g/L; Bacto yeast—5 g/L; NaCl—10 g/L) or in Minimal Medium (Glucose 1g/L; KH_2_PO_4_ 10g/L, sodium citrate 0.5 g/L, MgSO_4_.7H_2_O 0.1 g/L; (NH_4_)_2_ SO_4_ 1g/L), at pH 7.0 (NaOH) at 37 °C with gentle shaking. 

### 2.4. Cloning of Iuc-GFP Vector

The *Iuc* promoter sequence was produced using synthetic DNA strands produced by Eurofins Genomics. Single strands were annealed in SSC buffer (Sodium Chloride, 0.15 M, and Sodium Citrate, 15 mM at pH 7.0) in a thermocycler (95 °C for 10 min, slowly cooled to 35˚C (Δ = −1 °C/min)). Double strand formation was confirmed by running electrophoresis gel and using single non-annealed filaments as standards. DNA was quantified using Thermo Scientific™ NanoDrop™ Spectrophotometers. The obtained *Iuc* promoter was then digested with the restriction enzymes BglII and HindIII to generate ends cohesive with the accepting pCDNA3.1 GFP vector. pcDNA3.1(+) eGFP was digested with BglII and HindIII [23]. The digested vector was purified via electrophoresis on a 0.8% Low Melting agarose. Vector and the insert were ligated and transformed in chemically competent *E. coli* cells. Correctness of cloning and transformation was ensured via sequencing of the transformed DNA.

### 2.5. Spectrofluorimetric Measurement of GFP Expression

Aliquots (50 µL) of *Iuc*-GFP expressing *E. coli* culture were challenged for *Iuc* promoter activity and GFP expression in a 96 or 354-well black optiplate (Perkin Elmer, Waltham, USA). Intracellular GFP fluorescence was measured in a Perkin Elmer Envision 2105 Multiplate reader (Perkin Elmer) as already reported [24], using the inbuilt monochromator and with the following parameters: λ_exc_ = 488 nm, λ_ems_ = 509 nm, and monochromator cut off 360 nm. After the GFP measurement, aliquots were transferred in transparent Optiplate (Perkin Elmer, Waltham, USA) for nephelometric measurement (λ = 600 nm). This second measurement indicates the total number of bacterial cells in each well and was used for normalization. Normalized GFP expression is reported as the ratio between intracellular GFP fluorescence and absorbance of the culture at 600 nm ± SD. Statistical analysis and *p* value calculation was performed by means of GraphPad Prism 6.0.

### 2.6. LC-HRMS and Molecular Networking of the Extracts

LC-HRMS and molecular networking were performed according to [25] with slight modifications. A Thermo LTQ Orbitrap XL high-resolution ESI mass spectrometer coupled to an Agilent model 1100 LC system was used to perform LC-HRMS experiments. A 5-µm Kinetex C18 column (50 × 2.10 mm), maintained at room temperature, was eluted at 200 mL min^−1^ with H_2_O (supplemented with 0.1% HCOOH) and MeOH, using gradient elution. The gradient program was as follows: 10% MeOH for 3 min, 10%→100% MeOH for 15 min, 100% MeOH for 12 min. Mass spectra were acquired in positive ion detection mode. Data were collected in the data-dependent acquisition (DDA) mode, in which the fifth most intense ions of a full-scan mass spectrum were subjected to high-resolution tandem mass spectrometry (HRMS/MS) analysis. The *m/z* range for data-dependent acquisition was set between 150 and 2000 amu. HRMS/MS scans were obtained for selected ions with CID fragmentation, isolation width of 2.0, normalized collision energy of 35, Activation Q of 0.250, and activation time of 30 ms. Data were analysed using Thermo Xcalibur software. 

Pre-processing of raw files was performed through MZmine 2.53 [26] The mass detection was performed on raw data and exact masses with mass level 1 and centroided masses with mass level 2, by keeping the noise level at 10,000. Chromatograms were built using an ADAP module with a minimum height of 10,000, and *m/z* tolerance of 0.01 (or 20 ppm). Peak alignment was performed using the Join aligner algorithm (*m/z* tolerance at 0.01 (or 10 ppm), absolute RT tolerance at 0.3 min). [M+Na–H], [M+K–H], [M+Mg−2H], [M+NH_3_], [M-Na+NH_4_], [M+1, ^13^C], [M-^35^Cl+^37^Cl] ^+^, [M+^56^Fe-3H]^+^ adducts were filtered out by setting the maximum relative height at 100%. Peaks without associated MS/MS spectra were finally filtered out from the peak list. Clustered data were then exported to an mgf file for GNPS, while chromatographic data including retention times, peak areas, and peak heights were exported to a csv file. A Feature-Based Molecular Network [27,28] was generated on GNPS’s online platform [29], with the following parameters: the precursor ion mass tolerance was set to 0.05 Da and the MS/MS fragment ion tolerance to 0.05 Da, cosine score above 0.7 and more than 5 matched peaks. For GNPS’s library search, a cosine score of 0.7 and at least 6 matched peaks were set. The molecular network was visualized using Cytoscape software [30].

## 3. Results

### 3.1. Identification of the Promoter Region of the Operon Iuc in Anabaena Flos Aquae

We have previously reported that *Anabaena flos aquae* UTEX 1444 produces a cluster of chemically related siderophores (including schizokinen, rhizobactin 1021, synechobactin, and aerobactin) in a condition of iron limitation [31,32]. Till now, the yield of these siderophores from *Anabaena flos aquae* has been low [31]. Schizokinen is one of the most structurally well-defined hydroxamate siderophores and is produced by some *Anabaena* species in freshwater [33]. It is formed when two molecules of N4-hydroxy-1-aminopropane are bound to citrate at positions 1 and 3 of citric acid [5]. Schizokinen is an intermediate of synthesis of another siderophore, rhizobactin 1021, with whom it shares the same enzymatic producing machinery. This latter is also involved in the biosynthesis of the two structurally similar siderophores synechobactin and aerobactin [34,35]. 

**Figure 1:**
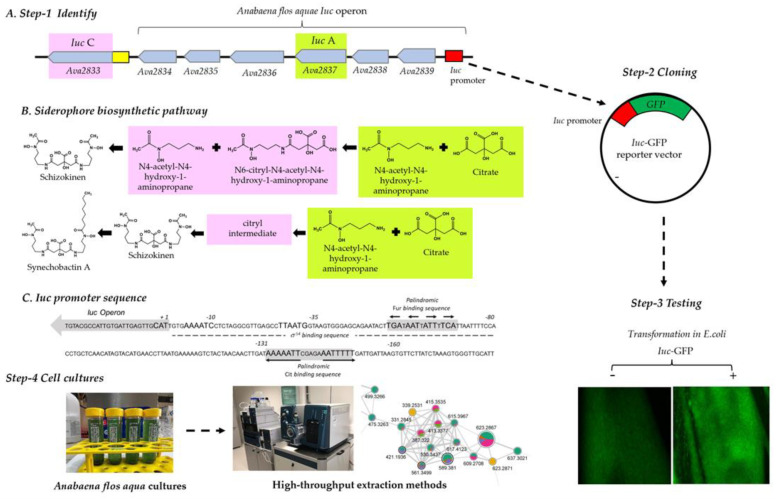
(**A**): Description of the four steps of the proposed platform: Step 1: Identification of the operon: the cartoon describes the arrangement of *Iuc* operon in *Anabaena flos aquae* UTEX 1444 (namely, *A. variabilis* ATCC 29413) with the *Iuc* promoter (red box), *IucA* (green region) and the *IucC* (pink region) genes highlighted; Step 2: Cloning of *Iuc* promoter (red box) upstream of the cDNA coding for GFP (green box) in the reporter vector *Iuc*-GFP; Step 3: testing of conditions activating GFP expression in transformed *Iuc*-GFP *E. coli.* The photographs show *E. coli* transformed (+) or not (-) with *Iuc*-GFP growing on agar plate and analysed under a fluorescent microscope; Step 4 analyses of *Anabaena flos aquae* cultures via dereplication by the combined use of high-resolution tandem mass spectrometry with molecular networking. (**B**) Biosynthesis reactions leading to schizokinen and synechobactin A with, highlighted in green and pink, those catalysed by *IucA* and *Iuc*, respectively. (**C**) *Iuc* promoter sequence showing the first ATG (+1) in the *Iuc* operon and the σ^54^ binding region. Predicted Fur and Cit binding sequences are highlighted in grey.

While siderophores are usually produced by non-ribosomal peptide synthetase (NRPS), schizokinen, rhizobactin 1021, synechobactin and aerobactin backbones are assembled by a NRPS-independent siderophore synthetase (NIS) [36]. The NIS biosynthesis pathway for schizokinen involves the proteins *IucA* and *IucC* (Figure 1A). *IucA* catalyses the attachment of the first N-acetyl-N-hydroxylysine to a carboxylic group of citric acid to yield N-citryl-N-acetyl-N-hydroxylysine, whereas *IucC* catalyses the attachment of the second N-acetyl-N-hydroxylysine to the carboxylic group of N-citryl-N-acetyl-N-hydroxylysine to yield schizokinen [37] (Figure 1A). 

The first step of our screening pipeline included the identification of NIS operon in the genome of *Anabaena flos aquae* UTEX 1444 (namely, *A. variabilis* ATCC 29413). GenBank allocates the *IucA/IucC* operon (from locus *Ava_2833* to locus *Ava_2839*, Figure 1A) in a ~12.5 Kb long sequence on the complementary strand of *Anabaena* chromosome (genomic region 3,506,499 to 3,518,085). The region was analysed with an Operon mapper [38] to identify operons, transcription units and promoter regions. All the transcripts present high similarity to proteins involved in siderophore biosynthesis, as reported in Supplementary Figure S1. The software attributes to the aminoacidic sequences coded by the genes *IucA*, *Ava_2836*, *Ava_2835* and *IucC* similarity to proteins involved in the rhizobactin siderophore biosynthesis *RhbC* (*IucA*), *RhbD* (Acetyltransferases, including N-acetylases of ribosomal proteins), *RhbE* (Lysine/ornithine N-monooxygenase) and *RhbF (IucC)*, respectively [39]. *Ava_2839* and *Ava_2838* code for an aminobutyrate aminotransferase and a Glutamate decarboxylase, respectively. *Ava_2834* was instead found to be similar to a hypothetical protein with unknown function. A single operon is predicted spanning from *Ava_2839* to *Ava_2834* and includes *IucA* but not *IucC,* which, on the contrary, seems to be an independent transcription unit (Figure 1A). The *Iuc* operon presents a ~670 bp long promoter region allocated upstream of the *Ava_2839* coding sequence (Figure 1C). The promoter was analysed in silico with the prediction tool BacPP [40]. The software confirmed the presence in the first 160 bp of the promoter of a binding region for sigma factors σ54 (promoting transcription of genes as consequence of metabolite deprivation) as well as a conserved palindromic binding sequence for Fur proteins [41] (inhibiting transcription of genes in the presence of iron) (Figure 1C). 

### 3.2. Construction of the Reporter Vector for Iuc Promoter Activity

A DNA fragment corresponding to the 160 bp long operon promoter region (genomic region 3,517,420 to 3,517,580) was synthesized in vitro and ligated into the vector pCDNA3.1(+) E-GFP, between restriction sites for the endonucleases BglII and HindIII, to create an expression vector (from now on, referred to as *Iuc*-GFP (Figure 1A, step 2)). The cloning procedure removed from the vector any other promoter sequence that could have influenced the bacterial expression of GFP. In order to facilitate the cloning process, two flanking regions cohesive to BglII and HindIII were included at the 5′ and 3′ ends of the *Iuc* promoter, respectively. The *Iuc* -GFP reporter vector was then transformed in *E. coli* for the downstream steps of the pipeline (Figure 1A, step 3). Correctness of the DNA sequence was confirmed upon sequencing of the full vector. Compared to untransformed *E. coli* cells, *Iuc*-GFP cells were endowed with a brighter intracellular green fluorescence, as shown in the microscopy panel of Figure 1A, suggesting a basal activity of the *Iuc* promoter in *E.coli*.

### 3.3. Screening of the Environmental Conditions Promoting Iuc Promoter Activation

The screening of the growth conditions stimulating *Iuc* promoter activity in *E.coli* cells was achieved by measuring *E. coli* intracellular GFP fluorescence. The multiplate Spectrofluorometer used to measure GFP intensity (see Methods for details) required very small culture volumes (50 μL) and allowed the simultaneous testing of multiple growth parameters with high reproducibility, as a result of the several technical and experimental replicates. We started testing environmental conditions known to stimulate production of secondary metabolites (often produced by bacteria in response to thermic, acid or osmotic stress conditions) as well as substrates of Iuc enzymes (citrate and Lysin). Table 1 reports the tested parameters, the range of testing, the optimal conditions suggested by the screening and the fold induction of GFP fluorescence achieved at the optimal condition compared to the normal growth condition. As result of the screening, we identified optimal GFP expression from *Iuc*-GFP in *E. coli* grown in Luria Broth Medium (as well as in Minimal Media): optimal temperature 29 °C, pH 7.5, and supplementation with Glucose (2g/L), NaCl (9.5 g/L), Fe^3+^ 39.5 μM (or 37 μM Fe^2+^), 2.6 mM Citrate and 0.75 mg/L of Lysine.

**Figure 2:**
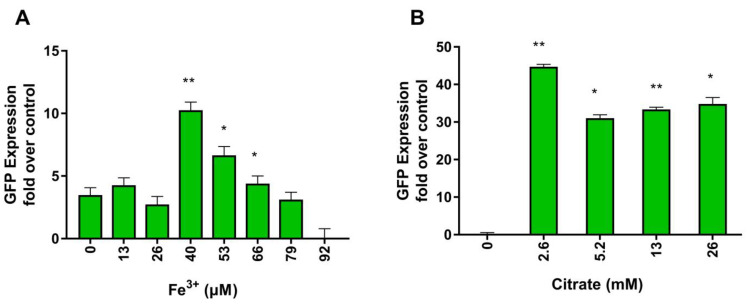
Activation of the *Iuc* promoter expressed as GFP expression from *Iuc*–GFP at the indicated Fe^3+^ (**A**) and citrate (**B**) concentrations. Fold induction refers to intracellular GFP fluorescence measured in *E. Coli* at the indicated conditions and normalized to GFP fluorescence measured in normal growth condition. Values are reported as mean + S.D. (n = 5 replicates, *p* value * < 0.05, ** < 0.01).

In cyanobacteria, the production of siderophore is upregulated under iron limitation. The *Iuc* operon is transcriptionally regulated by Fe^3+^ ions, thanks to cis-acting transcription repressors belonging to the Fur family [41]. In the presence of iron, Fur proteins bind and repress the *Iuc* operon. In a condition of iron limitation, Fur proteins detach from the promoter allowing cyanobacterial RNA polymerase to transcribe the operon. As shown in Figure 1C, detailed analysis of the promoter region of *Iuc* of *Anabaena flos aquae* confirmed in this strain the presence of the Fur responsive element. One of the strategies commonly used to increase the yield of in vitro produced siderophore is to cultivate cyanobacteria in iron-depleted media [31]. However, it is notoriously difficult to determine for each specific cyanobacteria the optimal Fe^3+^ concentration since iron is essential for many other bacterial processes. Indeed, as shown in Figure 2A and Table 1, in our recombinant system, the ability of iron to control the *Iuc* promoter of *Anabaena flos aquae* is preserved, with intracellular GFP expression depending on Fe^3+^ concentration. The relation between Fe^3+^ concentration and GFP production is described by a bell-shaped curve with optimal Fe^3+^ concentration for GFP in the micromolar range. 

Citrate is one of the substrates of *IucA*. As with many other substrates, it is a limiting factor for the activity of the enzyme. However, the results of Table 1 suggest that citrate can as well control the operon *Iuc* at the transcriptional level, and thus, regulates the expression of *IucA*. Indeed, as shown in Figure 2B and Table 1, increased concentrations of citrate stimulate *Iuc* promoter activity and augment GFP expression. There has already been reported the existence of citrate responsive elements in microbial genomes and the existence of regulators of citrate metabolism, like the proteins belonging to the family of Cit promoters [29] from *Enterococcus faecalis*. In the presence of citrate, Cit proteins bind to *cis*-acting sequences located upstream of genes and operons, usually those involved in citrate utilization. As shown in Figure 1C, detailed analysis of the promoter region of *Iuc* revealed the presence of a hypothetical citrate responsive element similar to those recruiting CitO.

### 3.4. Optimization of Culture Conditions of Anabaena Flos-Aquae UTEX1444 for Siderophore Production

To finally prove the eligibility of our four-step pipeline as a platform to identify the growth condition increasing the yield of siderophores production in *Anabaena flos aquae* UTEX 1444, we cultivated the strain in different media, modified in accordance with the screening results shown in Table 1. For siderophore identification and quantitation, we used MS-based molecular networking [27], a powerful metabolomic tool that allows a complete metabolic profiling of the species under study. MS-based molecular networking is a robust and less time-consuming bioinformatic tool for MS/MS data analysis and dereplication of complex natural matrixes. It assumes that structurally related compounds share similar fragmentation patterns and provides a visual representation of structural relationships between compounds in the extracts, as revealed by MS/MS data. The MS/MS spectra of the compounds in the extracts are aligned, the peaks in common between the various spectra are identified (the so-called peaks match), and based on the alignments, a score is assigned (cosine score). The higher the scoring, the more similar the spectra are. In a molecular network, each MS/MS spectrum is represented as a node, labelled with the parent mass, and the relatedness between compounds (cosine score) is represented by edges. Molecular networking presents high scalability and it allows an easy evaluation (in a single visual network) of multiple datasets, [42] avoiding a huge amount of complex data, always difficult to manage, as well as the capture and quantification of molecular analogues. Moreover, it enables a facilitated quantitative comparison of the desired metabolites between the samples, dispensing from the laborious peak-by-peak analyses, as in standard LC-MS data.

**Figure 3:**
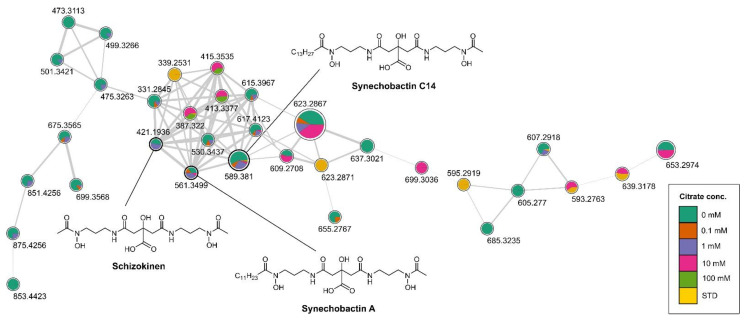
Cluster containing synechobactins in the molecular network obtained combining the LC-HRMS/MS analyses of all the extracts of #1-6 cultures of *Anabaena flos-aquae* UTEX 1444. Nodes are labelled with parent mass and are represented as a pie chart with colour coding showing the source culture of the compound ((Citrate) = 0 mM, 0.1 mM, 1 mM, 10 mM, 100 mM, STD, standard citrate concentration). Size of the nodes corresponds to the sum of precursor ion intensities. Edge thickness is related to cosine similarity score.

Differently from *E. coli*, *Anabaena flos aquae* UTEX 1444 cultures are usually maintained at 28 to 29 °C and the FWBG11growth medium has a pH of 7.5 and physiological osmolarity. This reveals that the temperature, pH and osmolarity at which this strain grows best already correspond to those leading to the highest transcription of the *Iuc* operon (according to Table 1). We thus maintained fixed these parameters and modified growth media varying Fe^3+^ and citrate concentration. Till now [31], the culture medium used for siderophore production was Fe^3+^deprived and contains 31 μmols/L of citric acid. We thus verified the effect of different iron and citrate concentration on the yields of siderophore produced. As first, six cultures (no added citrate, 0 mM (final (citrate) 31 μM, #1), 0.1 mM ((citrate) 131 μM, #2), 1 mM ((citrate) 1.031 mM #3), 10 mM ((citrate) 10.031 mM, #4), 100 mM ((citrate) 100.031 mM #5), STD ((citrate) 31 μM in the presence of Fe^3+^, #6) were extracted and analysed through LC-HRMS and molecular networking [31]. In the comprehensive molecular network of *Anabaena* #1-6 cultures’ extracts (Figure 3 and Figure S2) [43], the arrangement of the cluster containing schizokinen and the known synechobactins A and C14 showed an uneven distribution of such siderophores in the different cultures, with a clear prevalence in those with the lowest concentrations of citrate (31 to 131 µmol of citrate/L) and in the absence of Fe^3+^. 

**Figure 4:**
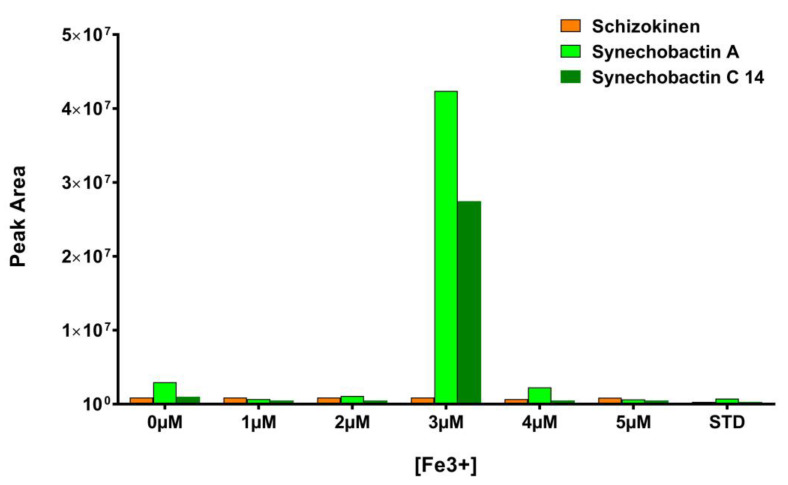
LC-HRMS quantitation of schizokinen (*m/z* 421.1936, orange bars), synechobactin A (*m/z* 561.3499, green bars) and synechobactin C14 (*m/z* 589.3810, blue bars) in *Anabaena flos-aquae* UTEX1444 cultures (FWBG) containing 0 μM, 1 μM, 2 μM, 3 μM, 4 μM, 5 μM and 23 μM (STD condition) Fe^3+^. (Representative of three experiments.)

Combining this information with those coming from our previous study [31], a new set of cultures was prepared, in which the concentration of Fe^3+^, supplemented as ferric ammonium citrate in FWBG11, has been varied from 0 µM to 5 μM. Taking into account that µM quantities of citrate are necessary to promote synechobactin production (Figure 3), sodium citrate has been supplemented to each of the above cultures in the amount necessary to compensate for the citrate removed with the ferric ammonium salt. The extracts of the above cultures were subjected to LC-HRMS. Figure 4 shows the results of this analysis: at a concentration of 3 μM Fe^3+^, the production of synechobactin A and synechobactin C14 is sharply increased, confirming the bell-shaped correlation between the amount of Fe^3+^ and siderophore production suggested by our in vitro pipeline. 

## 4. Discussion

Siderophores, bioactive molecules able to chelate iron, are secreted in the environment by microorganisms for iron intracellular uptake and maintenance of cellular homeostasis. They can be used as a greener and sustainable tool for the bioremediation of contaminated ecosystems, as well as in drug discovery. 

Scientific advances in culture media formulation and cultural conditions affecting the production of siderophores have been recently summarized by Soares [44]. Optimization of the growth conditions is time consuming, especially for a slow growing microorganism. Siderophores are usually produced and secreted in micromolar quantities with yields affected by different environmental parameters, including iron concentration, carbon and nitrogen source, temperature and aeration [45]. When achieved, optimization has allowed the successful production of siderophore in bioreactors, using Batch Fermentation [46]. 

Most of the reports available in the literature describe optimization procedures including indirect identification and quantitation of siderophores either via the Chrome Azurely S (CAS) assay or via growth inhibition of iron-dependent parasites by optimized siderophore conditioned media. Despite the difficulty related to optimization, the increased yield of produced siderophores allows, on the one hand, downstream application of these biomolecules, and also reveals new aspects underpinning microbial control of siderophore intracellular production. Nasr Ghazy and Sahar El-Norway were able to optimize siderophore production in six strains of rhizobacteria, measuring the ability of conditioned media to inhibit the growth of the iron-dependent parasite *C. maydis* in *Zea Mayis* [47], interestingly, achieving yield promotion of maize plant under laboratory, greenhouse and field conditions. Sasirekha and Srividya [48] optimized siderophore production in cultures of *P. aeruginosa* using CAS agar plate and proved that metals other than Fe^3+^ are able to affect yields of siderophore production. Siderophore production was optimized using a microplate CAS shuttle assay also in *P. fluorescens*, *P. putida*, *B. stabilis* and *O. oryzae* by Murakami et al. [49] *P. fluorescens* DSM 50090, an industrial relevant strain for siderophore production, was found to produce a high amount of siderophore in a minimal medium containing succinate [50]. Recently, Lemare et al. engineered *P. aeruginosa* to obtain a strain able to produce the siderophore Pioverdin, even in the presence of iron. This iron-insensitive siderophore-producing mutant will find application in asbestos weathering [51]. 

While optimization of siderophore production has been achieved in different bacterial strains, scarce information is available on optimization in cyanobacteria. Here, we propose an alternative four-step platform to study and optimize at the transcriptional level growth conditions to increase the production of siderophores in *Anabaena*. The pipeline is here validated using the reference strain *Anabaena flos-aquae* UTEX 1444 that was previously shown to produce a cluster of chemically related siderophores (including schizokinen, rhizobactin, synechobactin and aerobactin) in a condition of iron (III) deprivation [31]. 

Our pipeline offers different advantages compared to the traditional OSMAC approach. The first is the time required for identifying factors affecting siderophore production. Considering the growth time (30 days) of the strain and the necessary culture volume required to yield enough biomass of *Anabaena flos aquae* (at least 50 mL)*,* the OSMAC strategy is time-consuming and expensive. Here, using smaller culture volume (50 μL) and fast-growing bacteria, our pipeline allowed the fast identification of temperature, pH, osmolarity, iron and citrate concentration as growth factor influencing, at a transcriptional level, the production of siderophore, i.e., schizokinen and synechobactin A and C14. Other advantages of our pipeline include its low cost, its amenability to the high-throughput pipeline and its applicability to a greater number of cyanobacteria. 

Furthermore, the use of molecular networking, as a fourth step of the platform, has proved successful in the direct and unambiguous identification of siderophores and their quantification and more rapid than traditional techniques (LC-MS, CAS).

Finally, the pipeline was able to reveal unprecedented aspects of the siderophore transcriptional regulation in *Anabaena flos aquae*. By identifying citrate (a substrate of siderophore production) as a growth factor influencing at the transcriptional level siderophore production, the pipeline allowed the identification of a citrate responsive element allocated upstream of the *Iuc* promoter region, giving more insight into the genetic regulation of siderophore production in cyanobacteria and probably in other microorganisms. 

## Figures and Tables

**Table 1:** Results of the optimization of GFP expression from the reporter vector *Iuc*-GFP in *E. coli*.

	Luria Broth (LB) Medium		
Parameter	Range Tested	Optimal Value	Fold Induction at Optimal Value ^1^
Temperature (°C)	23–37	29	9.1 ± 0.1 *
pH	6.0–8.0	7.5	2.7 ± 0.8 **
NaCl g/L	8.0–10.0	9.5	2.8 ± 0.4 **
Fe^3+^ (μM)	0–52	37	41.3 ± 0.9 **
Fe^2+^ (μM)	0–92	39.5	6.5 ± 0.1 **
Citrate (mM)	0–52	2.6	44.7 ± 0.1 **
Glucose (g/L)	0–10	2	25.9 ± 0.3 *
Lysine (mg/L)	0–5	0.75	5.3 ± 0.4 **
	**Minimal Medium**		
**Parameter**	**Range tested**	**Optimal value**	**Fold Induction at optimal Value ^1^**
Temperature (°C)	23–37	29	9.2 ± 0.8 *
pH	6.0–8.0	7.5	3.3 ± 0.8 **
NaCl g/L	8.0–10.0	9.5	2.2 ± 0.2 *
Fe3+ (μM)	0–52	30	20.0 ± 0.1 **
Fe2+ (μM)	0–92	92	20.7 ± 0.1 **
Citrate (mM)	0–52	2	8.3 ± 0.1 **
Glucose (g/L)	0–10	10	43.6 ± 0.1 **
Lysine (mg/L)	0–5	0.75	3.1 ± 0.1 **

^1^ fold induction at optimal value = intracellular GFP fluorescence measured in *E. coli* at the indicated optimal condition and normalized to GFP fluorescence measured in normal growth condition (supplemented with LB Medium or Minimal Medium). Values are reported as mean + S.D. (n = 5 replicates, *p* value * < 0.05, ** < 0.01).

## Data Availability

The data presented in this study are available at https://gnps.ucsd.edu/ProteoSAFe/status.jsp?task=53fbad6b425540ceb5eae088aac6ae32.

## Supplementary Materials

The following supporting information can be downloaded at: https://www.mdpi.com/article/10.3390/metabo13020154/s1, Figure S1: Putative enzymatic activities and biosynthetic pathway of schizokinen; Figure S2: LC-HRMS/MS analyses of *Anabaena flos aquae* extracts.
