# Supplementary material for: A Four-Step Platform to Optimize Growth Conditions for High-Yield Production of Siderophores in Cyanobacteria
Source: Metabolites. 2023 Jan 20;13(2):154. doi: 10.3390/metabo13020154 (PMC9967094; doi:10.3390/metabo13020154)
Supplement: Supplementary file 1 [file metabolites-13-00154-s001.zip › metabolites-2117725-supplementary.pdf]

Supplementary Materials

A four-step platform to optimize growth conditions for high-yield production of siderophores in Cyanobacteria

Karishma Kundu[1,2], Roberta Teta[1], Germana Esposito[1], Mariano Stornaiuolo[2]*& Valeria Costantino[1]

[1]The Blue Chemistry Lab, Department of Pharmacy, University of Naples Federico II, Via Domenico Montesano 49, 80131 Napoli, Italy
[2]The BioMol Lab, Department of Pharmacy, University of Naples Federico II, Via Domenico Montesano 49, 80131 Napoli, Italy
*Correspondence: mariano.stornaiuolo@unina.it

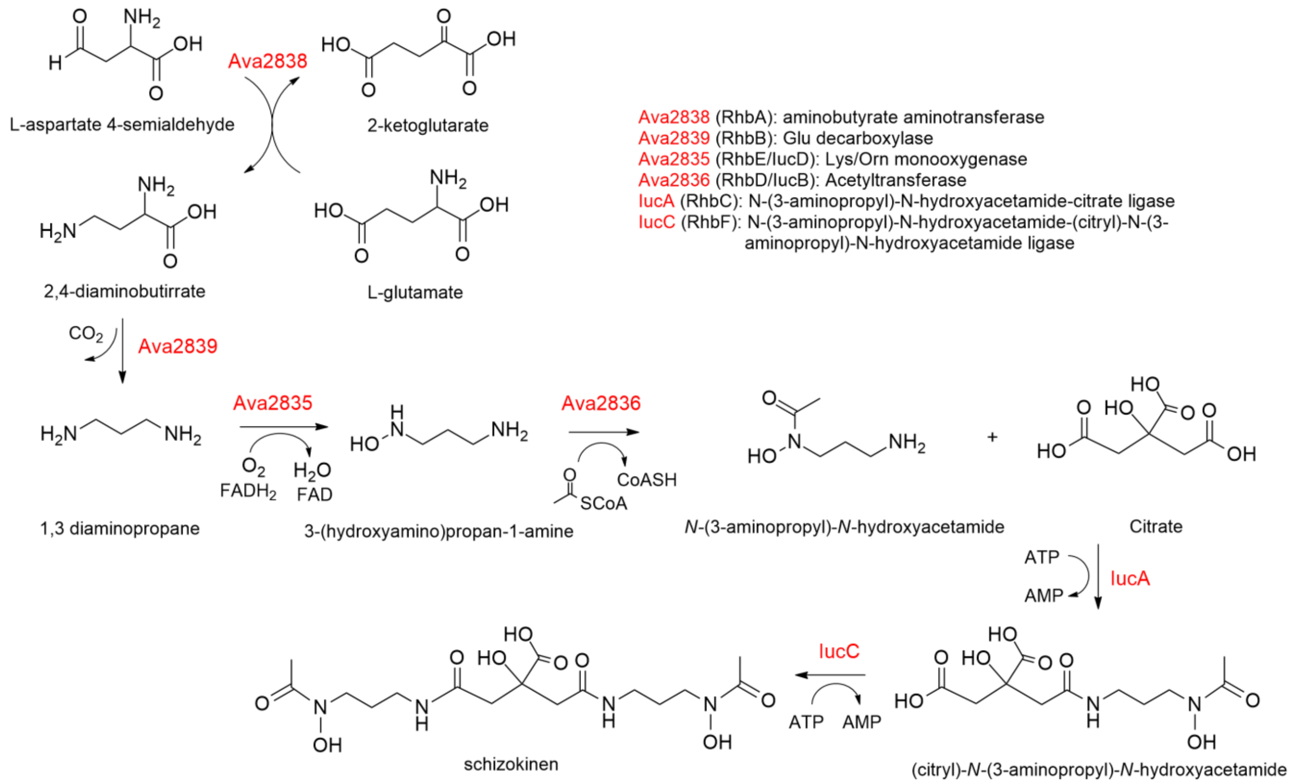

Figure S1. Putative enzymatic activities and biosynthetic pathway of schizokinen.

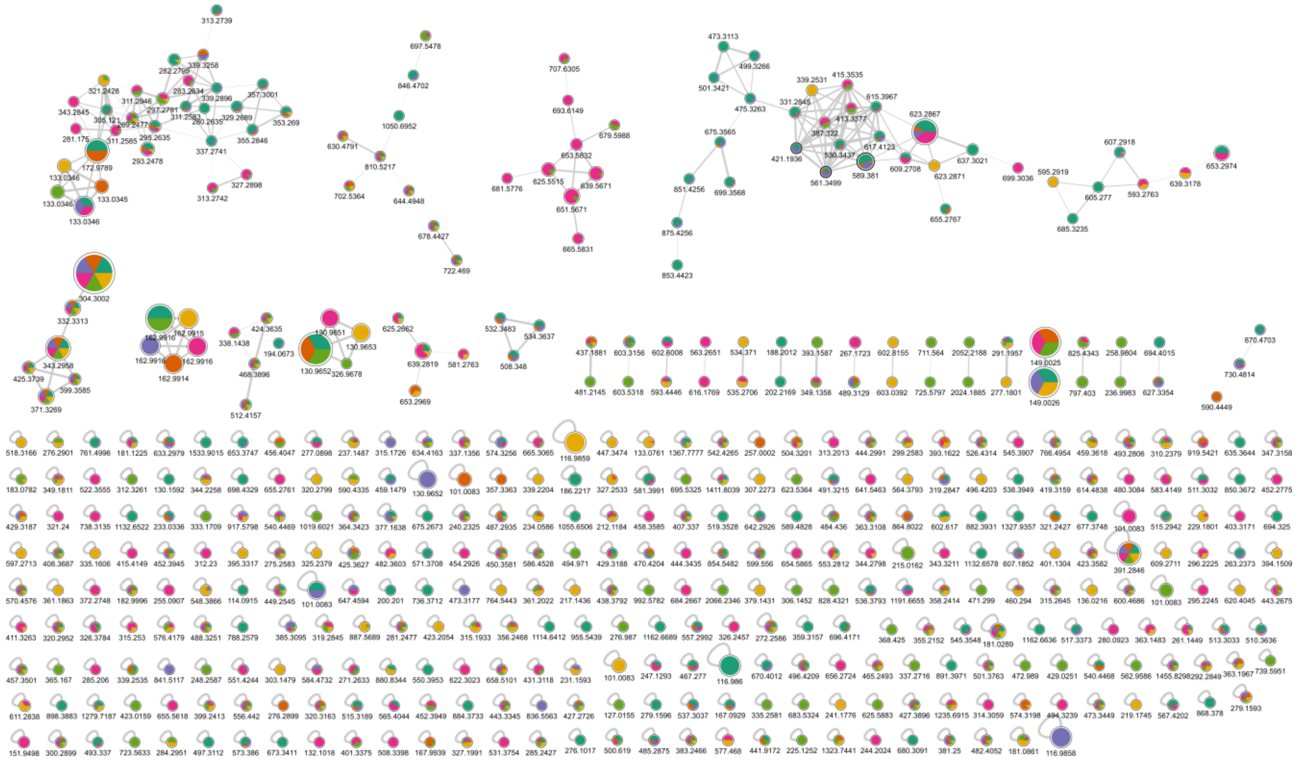

Figure S2. LC-HRMS/MS analyses of *Anabaena flos aquae* extracts. The molecular network obtained combining the LC-HRMS/MS analyses of all the extracts of *#1-6* cultures of *Anabaena flos-aquae* UTEX 1444. Nodes are labeled with parent mass and are represented as a pie chart with color coding showing the source culture of the compound ([Citrate] = 0 mM, 0.1 mM, 1 mM, 10 mM, 100 mM, STD, standard citrate concentration). Size of the nodes corresponds to the sum of precursor ion intensities.
